# Association between serum vitamin D and depressive symptoms among female workers in the manufacturing industry

**DOI:** 10.1186/s40557-015-0083-y

**Published:** 2015-12-12

**Authors:** Soon Il Kwon, Jun Seok Son, Young Ouk Kim, Chang Ho Chae, Ja Hyun Kim, Chan Woo Kim, Hyoung Ouk Park, Jun Ho Lee, Jun Ick Jung

**Affiliations:** Department of Occupational and Environmental Medicine, Samsung Changwon Medical Center, Sungkyunkwan University School of Medicine, Changwon City, Republic of Korea

## Abstract

**Background:**

Vitamin D has been known to maintain the body’s balance of calcium and phosphorus as well as skeletal health. There has been increasing emphasis on the importance of vitamin D as recent studies have been reporting the specific functions of vitamin D in the cerebral nervous system and the association between the level of serum vitamin D and depressive symptoms. However, there is currently a paucity of research investigating the association between serum vitamin D and depressive symptoms in Korean subjects. Consequently, this study has aimed to determine the level of serum vitamin D and explore the association between serum vitamin D and depressive symptoms in Korean female workers.

**Method:**

A medical examination, questionnaire, anthropometric measurements, and a blood test were conducted between February 3 and March 7, 2014 in 1054 subjects among female workers in the manufacturing industry who underwent physical examinations in a university hospital. From this data, we identified the level of serum vitamin D and investigated the association between serum vitamin D deficiency and depressive symptoms.

**Results:**

The average serum vitamin D level of the 1054 subjects was 9.07 ± 3.25 ng/mL, and the number of subjects in the serum vitamin D deficiency group with less than 10 ng/mL was 721 (68.4 %). The odds ratio of the depressive symptom group with a CES-D score of 16 or above being in the deficiency group with a serum vitamin D level less than 10 ng/mL was found to be 1.55 (95 % CI = 1.15–2.07).

**Conclusion:**

68.4 % of female workers in the manufacturing industry were in the deficiency group with serum vitamin D levels less than 10 ng/mL. Additionally, we identified an association between serum vitamin D deficiency and depressive symptoms. In the future, if serum vitamin D deficiency is checked regularly in workers, we expect to achieve better outcomes in managing their depressive symptoms.

## Background

Vitamin D is activated in the skin through sun exposure and is well known to be involved in maintaining the balance of calcium and phosphorus as well as in skeletal health [1]. Recently, studies have reported newly discovered functions and mechanisms of vitamin D, such as cell proliferation and regulation of cell division [2], regulation of immune functions [3], and antitumor activity [4]. Additionally, with studies reporting the association of vitamin D deficiency with high blood pressure [5], diabetes [6], obesity [7], and cardiovascular disease mortality [8], the importance of vitamin D has gained increasing attention.

According to the study on the prevalence of serum vitamin D deficiency in Koreans using the data of the Korea National Health and Nutrition Examination Survey (KNHANES 2010–2011), only 28.3 % of the subjects who were 10 years of age or above were in the normal range (20 ng/mL or above), and in males and females, 34.2 and 22.4 %, respectively, were in the normal range [9]. In a study of Korean wage workers, although 29.2 % of women between 50 and 69 years were in the normal range (20 ng/mL or above), only 8.4 % of women between 20 and 29 years were in the normal range [10]. Attention in the importance of vitamin D is increasing; nevertheless, the problem of serum vitamin D deficiency in Koreans is clearly severe, especially in the younger female generations. Additionally, in a study on the association between shift work and bone mineral density, the level of serum vitamin D in shift workers was found to be significantly lower than that of day workers [11], which implies a serious level of serum vitamin D deficiency in Korean female workers.

Depression is a mood disorder whose major symptoms are feelings of sadness, loneliness, and loss of motivation [12]. The World Health Organization (WHO) has reported depression to be the third most prevalent disorder out of the ten leading contributors to the Global Burden of Disease [13]. According to Korea Health Statistics in 2013, the prevalence of depressive symptoms, that is, feelings of sadness or despair that affected daily activities for two or more consecutive weeks in the past year was 10.3 % [14]. According to the Epidemiological Survey of Mental Disorders conducted every 5 years by the Ministry of Health and Welfare enacted by the Mental Health Act in 2001, the lifetime prevalence rate of major depressive order increased from 4.0 % in 2001, to 5.6 % in 2006, and eventually 6.7 % in 2011 [15]. Additionally, as depression is a strong risk factor of suicide [16, 17], Korea ranks first among the Organization for Economic Cooperation and Development (OECD) countries with a suicide rate of 28.5 per 100,000 people [18], which is rather high in comparison to the OECD average of 12.1 per 100,000 [19]. The increasing lifetime prevalence of major depressive disorder may be a latent problem as a causative agent of suicidal behaviors, and depression is reported to have significant correlations with increase of absence in workers, decrease of work competence, decrease of productivity, and increase of health expenditures [20], suggesting that depression is not only a mental burden to an individual, but also a problem in terms of the health management of workers and an economic burden. Because women especially are known to have a 2 ~ 3-fold greater probability of experiencing depression during their lifetime than men [21], depression is a critical problem in the health management of Korean female workers.

Recent studies have reported the association between serum vitamin D and depression. In a cross-sectional study in Britain [22], a negative correlation was identified between the serum vitamin D level and depressive symptoms, and in the prospective cohort study in the United States [23], it was found that serum vitamin D levels were associated with incident depression. As vitamin D is reported to have specific functions in the central nervous system, it is also known to be associated with mental disorders including depression [24].

The high prevalence of serum vitamin D deficiency in Korean females [9], the significantly low serum vitamin D concentration in shift workers compared to day workers [11], the increasing lifetime prevalence of depressive disorder in Korea [15], and the higher susceptibility to depression in females than males are all latent problems in the health management of Korean female workers. Furthermore, few published studies have investigated the association between serum vitamin D and depressive symptoms in Korean subjects. Consequently, this study has aimed to determine the level of serum vitamin D and explore the association between serum vitamin D and depressive symptoms in Korean female workers.

## Methods

### Subjects

This study is a cross-sectional study using data from health examinations. Among female electronics manufacturing indoor workers, the subjects of this study were 1646 female examinees living in the city of Busan and the adjacent Gyeongnam area who underwent a health examination in the provincial capital of Changwon between February 3 and March 7, 2014. Seven females with psychiatric history including depression, and 13 females who were divorced or separated after marriage were excluded. After eliminating subjects lacking examination results and data, the total number of subjects was 1054 females. All participants in this study signed an informed consent form. This study was reviewed by the institutional review board (IRB) of the university hospital before implementation (IRB No. 2015-SCMC-023-00).

### Measurement of serum vitamin D

To determine the level of serum vitamin D, blood 25-hydroxyvitamin D (25(OH)D) samples were cryopreserved and measured by electrochemiluminescence immunoassay using the Modular E (Hitachi Co, Tokyo, Japan) device. Blood 25-hydroxyvitamin D (25(OH)D) can represent a value by both supply routes activated in the skin through sun exposure and obtained by food [1]. The subjects were classified into the low group or high group relative to the standards of deficiency defined as 10 ng/mL in previous studies [25].

### Level of depression

To assess the level of depression, the Korean version of the Center for Epidemiologic Studies Depression Scale (CES-D), a self-report depression scale developed by the US National Institute of Mental Health was used. Since the CES-D is a commonly used tool to identify depressive symptoms in the general population, it is known to be a reliable tool in differentiating between people with and without depressive symptoms [26]. The tool has a total of 20 items and a maximum score of 60, and each item is scored on a scale from 0 to 3 according to the frequency of a given depressive symptom experienced during the past week, with a higher score, indicating a higher level of depression. The cut-off value is usually 16 and 25, with 16 being probable depression and 25 being definite depression [27]. In this study, the cut-off value of 16 was used to divide the subjects into a group with a score under 16 and a group with a score of 16 or above.

### Definition of other variables

To assess sociodemographic characteristics, the age, level of education, and body mass index (BMI) were investigated, and to assess lifestyle, smoking, drinking, and regular exercise were investigated. To assess occupational factors, whether the subject had shift work or not was determined. The subjects were divided by age into a group less than 30 years of age and a group 30 years of age and above, and they were also classified by marital status into a single group and a married group. Level of education was distinguished by those with a high school diploma or less and those with a college diploma or above. The subjects were classified by BMI into an underweight group with a BMI less than 18.5, a normal weight group with a BMI 18.5–24.9, and an obese group with a BMI above 24.9, using the criteria of the WHO for the Asia-Pacific region. The subjects were divided by smoking status into a current smoker group and a non-smoker and ex-smoker group, and using the risky drinking classification of the National Health and Nutrition Examination Survey, the subjects were classified into a risky drinking group if they consumed five alcoholic drinks or more at least twice a week. The subjects were divided into a group that exercised at least three times a week and a group that exercised less than three times a week regardless of the intensity. Finally, the subjects were divided into a shift work group if they had a night shift from 10 p.m. to the next morning at 6.a.m. at least four times per month on average or worked an average of at least 60 hours per month during the night shift.

### Statistical analysis

To assess the serum vitamin D level in subjects, the independent samples *t*-test was used to determine the average serum vitamin D level according to the variables, while to determine the difference in distribution of serum vitamin D according to the variables, the chi-squared test was used. To investigate the influence of the variables associated with a significant difference in the distribution of the depressive symptom group, univariate logistic regression analysis was conducted, and after controlling for the variables with significant correlation, multivariate logistic regression analysis was conducted. We used IBM SPSS Statistics for Windows version 21 (IBM Corp., Armonk, NY, USA) for statistical analysis, and the confidence interval was set at 95 %, and the statistical significance at *P* < 0.05.

## Results

### Serum vitamin D level in the subjects

The average serum vitamin D level of the whole group of subjects was 9.07 ± 3.25 ng/mL. The value of subjects in the serum vitamin D deficiency group (*n* = 721; 68.4 %), that is, those with a level of less than 10 ng/mL, constituted a larger proportion of the subjects than the value (*n* = 333; 31.6 %) with 10 ng/mL or more of vitamin D. The average serum vitamin D level by age group was 8.86 ± 3.12 ng/mL in the group under 30 years of age and 9.61 ± 3.54 ng/mL in the group 30 years and above, and the value of subjects in the deficiency group (less than 10 ng/mL) was 545 (71.2 %) in the group less than 30 years and 176 (60.9 %) in the group 30 years and above, which shows that the distribution of the serum vitamin deficiency group was significantly skewed toward the group below age 30 (*p* = 0.001). The average serum vitamin D level according to shift work was 9.94 ± 3.25 ng/mL in the day worker group and 8.89 ± 3.23 ng/mL in the shift worker group; the value of subjects in the serum vitamin D deficiency group was 102 (56.0 %) in the day worker group and 619 (71.0 %) in the shift worker group, showing a significantly higher distribution of the serum vitamin D deficiency group in the shift worker group (*p* < 0.001). Regular exercise, current smoking, high-risk drinking, and BMI did not have significant relationship to the distribution of the serum vitamin D deficiency between groups (Table 1).

**Table 1:** Vitamin D serum concentration according to variables

Variables		Vitamin D (ng/mL) Mean (S.D)^b^		Vitamin D				
				≥10 ng/mL		<10 ng/mL		*p*-value^a^
				N	%	N	%	
Total		9.07	3.25	333	(31.6)	721	(68.4)	
Age (years)	<30	8.86	3.12	220	(28.8)	545	(71.2)	0.001
	≥30	9.61	3.54	113	(39.1)	176	(60.9)	
Shift work	Yes	8.89	3.23	253	(29.0)	619	(71.0)	<0.001
	No	9.94	3.25	80	(44.0)	102	(56.0)	
Regular exercise (times per week)	≥3	9.09	4.12	47	(31.8)	101	(68.2)	0.963
	<3	9.06	3.09	286	(31.6)	620	(68.4)	
Smoking habit	Current	8.81	3.21	47	(25.7)	136	(74.3)	0.058
	Never/former	9.12	3.26	286	(32.8)	585	(67.2)	
Risky drinking^c^	Yes	9.56	3.25	63	(38.0)	103	(62.0)	0.055
	No	8.97	3.25	270	(30.4)	618	(69.6)	
Body Mass Index (kg/m^2^)	<18.5	8.32	2.94	34	(24.5)	105	(75.5)	0.125
	18.5–24.9	9.15	3.34	238	(32.6)	493	(67.4)	
	>25	9.31	3.07	61	(33.2)	123	(66.8)	

^a^comparison by chi-squared test

^b^comparison by Student’s t-test

^c^Risky drinking: two or more times per week and five or more glasses each time

### Distribution of depressive symptoms according to general characteristics and the serum vitamin D level

The CES-D depression scale score of all the subjects taken together was 13.8, and the proportion of the depressive symptom group with a score of 16 or above was 33.9 %. The age level of the subjects ranged from 19 to 37, and the average age was 26.5 years. The percentage of the younger age group was 72.6 %. The distribution of the depressive symptom group was significantly skewed toward the group less than 30 years old compared to the group 30 years or above (*p* = 0.004). 82.7 % of the subjects were shift workers, and the distribution of the depressive symptom group was significantly skewed toward the shift worker group over the day worker group (*p* < 0.001). 81.4 % of the subjects were single, and those in the depressive symptom group were significantly skewed toward being single rather than married (*p* = 0.004). 67.6 % of the subjects had a high school education or less, and the distribution of depressive symptoms was significantly skewed toward this group over the group with a college diploma or above (*p* < 0.001). 14.0 % of the subjects had regular exercise (at least 3 times a week), 17.4 % were current smokers, and 15.7 % were high-risk drinkers. In terms of BMI, 13.1 % of the subjects were underweight, 69.4 % were within the normal range, and 17.5 % were overweight. There was no significant difference in the proportion of those with depressive symptoms according to the classifications of regular exercise, smoking habit, risky drinking, or BMI. The depressive symptom group comprised 26.1 % of the high serum vitamin D group and 37.4 % of the serum vitamin D deficiency group, which indicates that the depressive symptom group skewed significantly toward the serum vitamin D deficiency group (*p* < 0.001) (Table 2).

**Table 2:** Distribution of depressive symptoms

Variables		Number (%)		CES-D^a^				
				<16		≥16		*p*-value^b^
				N	(%)	N	(%)	
Total (N)				697	(66.1)	357	(33.9)	
Age (years)	<30	765	(72.6)	486	(63.5)	279	(36.5)	0.004
	≥30	289	(27.4)	211	(73.0)	78	(27.0)	
Shift work	Yes	872	(82.7)	550	(63.1)	322	(36.9)	<0.001
	No	182	(17.3)	147	(80.8)	35	(19.2)	
Marital status	Married	196	(18.6)	147	(75.0)	49	(25.0)	0.004
	Unmarried	858	(81.4)	550	(64.1)	308	(35.9)	
Education level	≥college	341	(32.4)	253	(74.2)	88	(25.8)	<0.001
	≤high school	713	(67.6)	444	(62.3)	269	(37.7)	
Regular exercise	≥3	148	(14.0)	96	(64.9)	52	(35.1)	0.726
(times per week)	<3	906	(86.0)	601	(66.3)	305	(33.7)	
Smoking habit	Current	183	(17.4)	111	(60.7)	72	(39.3)	0.085
	Never/former	871	(82.6)	586	(67.3)	285	(32.7)	
Risky drinking^c^	Yes	166	(15.7)	111	(66.9)	55	(33.1)	0.827
	No	888	(84.3)	586	(66.0)	302	(34.0)	
Body Mass Index (kg/m^2^)	<18.5	139	(13.1)	89	(64.0)	50	(36.0)	0.124
	18.5 ~ 24.9	731	(69.4)	497	(68.0)	234	(32.0)	
	>25	184	(17.5)	111	(60.3)	73	(39.7)	
Vitamin D	≥10	333	(31.6)	246	(73.9)	87	(26.1)	<0.001
(ng/mL)	<10	721	(68.4)	451	(62.6)	270	(37.4)	

^a^the Center for Epidemiologic Studies Depression Scale

^b^comparison by chi-squared test

^c^Risky drinking: two or more times per week and five or more glasses each time

### Association between serum vitamin D deficiency and depressive symptoms

To investigate which variables had a significant relationship to the depressive symptom group, logistic regression analysis was conducted. In the univariate logistic regression analysis, vitamin D deficiency showed a significant correlation with depressive symptoms (OR = 1.69, 95 % CI = 1.27–2.26). There were also significant direct correlations between the depressive symptom group and the night shift work group (OR = 2.46, 95 % CI = 1.66–3.64), lower education level group (OR = 1.74, 95 % CI = 1.31–2.32), younger age group (OR = 1.55, 95 % CI = 1.15–2.09), and single marital status group (OR = 1.68, 95 % CI = 1.18–2.39). In the multivariate logistic regression analysis, adjusting for variables found to be significant in the univariate logistic regression analysis, the correlation with the depressive symptom group in serum vitamin D deficiency group remained significant (OR = 1.55, 95 % CI = 1.15–2.07). The correlations with the depressive symptom group in the night shift work group (OR = 1.83, 95 % CI = 1.18–2.82) and lower education level group (OR = 1.39. 95 % CI = 1.02–1.90) also remained significant, but no significant correlations with age or marital status were found in the multivariate logistic regression analysis (Table 3).

**Table 3:** Univariate and multivariate logistic regression analysis of factors affecting depressive symptoms

Variables		Unadjusted		Adjusted^a^	
		OR^b^	95 % CI^c^	OR^b^	95 % CI^c^
Vitamin D	≥10	1.00		1.00	
(ng/ml)	<10	1.69	1.27–2.26	1.55	1.15–2.07
Shift work	No	1.00		1.00	
	Yes	2.46	1.66–3.64	1.83	1.18–2.82
Education level	≥college	1.00		1.00	
	≤high school	1.74	1.31–2.32	1.39	1.02–1.90
Age (years)	≥30	1.00		1.00	
	<30	1.55	1.15–2.09	1.15	0.82–1.62
Marital status	Married	1.00		1.00	
	Unmarried	1.68	1.18–2.39	1.19	0.80–1.77

^a^adjusted for vitamin D serum concentration, shift work, educational level, age, marital status

^b^odds ratio

^c^confidence interval

## Discussion

In this study, we determined the level of serum vitamin D in female workers in the manufacturing industry. The average serum vitamin D level in the subjects was 9.07 ± 3.25 ng/mL. Upon applying the serum vitamin D criteria used in previous studies [25] (deficiency defined as less than 10 ng/mL, insufficiency defined as between 10 ng/mL and 20 ng/mL, and optimum defined as 20 ng/mL and above), 68.4 % of the total subjects in this study fell into the deficiency group, while the insufficiency and deficiency groups together, that is, those who did not satisfy the optimal level of 20 ng/mL, comprised 97.2 % of the subjects. Because the vast majority of the subjects fell below the optimal level of 20 ng/mL, we split the subjects into a deficiency group with a cut-off of 10 ng/mL and a “high vitamin D” group of 10 ng/mL or above. Through this study, we were able to show that the prevalence of serum vitamin D deficiency and insufficiency was extremely high in this group of Korean female workers.

When comparing similar studies that have assessed the level of serum vitamin D in Korean female subjects, the average serum vitamin D concentration in the study of postmenopausal female subjects was 16.0 ng/mL and 76.0 % of the subjects fell into the insufficiency and deficiency group with a serum vitamin D level of less than 20 ng/mL [28]. The average serum vitamin D concentration in the study of female subjects between 19 and 39 years was 13.4 ng/mL, and 91.6 % of the subjects were in the insufficiency and deficiency group with less than 20 ng/mL [29]. The average serum vitamin D level and large proportion of subjects in the insufficiency and deficiency group (those with less than 20 ng/mL of serum vitamin D) in this study show great differences from the same measurements in the study on postmenopausal female subjects, and relatively little difference from the findings of the study with young female subjects. There are three plausible explanations for this. The proportion of young subjects in this study was high, as 72.6 % of the total subjects were females in their 20’s. A previous study reported that serum vitamin D deficiency is more severe in younger age groups than older age groups [10]. Likewise, in this study, the group aged less than 30 years showed a lower average serum vitamin D level than the age group 30 years and above, and the percentage of serum vitamin D deficiency was also significantly higher in the younger group. Additionally, the young female group may have had fewer opportunities for sun exposure through outdoor activities and have had a high rate of sunscreen usage [30, 31]. Second, 82 % of all the subjects in this study were shift workers. In a study of the association between shift work and bone mineral density [11], the shift worker group was found to have a lower serum vitamin D level than the day worker group, and in another study on the association between occupational factors and the serum vitamin D level [10], the shift worker group had a higher percentage of serum vitamin D deficiency. Similarly, in this study, the shift worker group had a lower average serum vitamin D level than the day worker group, and the percentage of serum vitamin D deficiency was significantly higher in the shift workers. Third, the serum vitamin D concentration was measured between February 3 and March 7, 2014. In previous studies, the serum vitamin D concentration has been lower in winter than in summer [29, 32], and thus considering the duration of this study, the amount of sunlight must have been less than during the summer.

In this study, the average score on the CES-D was 13.8, and 34 % of the subjects fell into the depressive symptom group. Several previous studies have also used the CES-D as a depressive symptom assessment tool with a cut-off value of 16 in Korean female subjects. In a study of 105 female police officers, 18 % of the subjects were in the depressive symptom group [33], while in the study of 2366 female subjects between 18 and 92 years after the Korean Financial Crisis of late 1997, 41 % of the subjects were in the depressive symptom group [34]. In this study, the percentage of subjects in the depressive symptom group was higher than in the study of female police officers, but lower than in the study of the general female population after the Korean Financial Crisis. Nevertheless, considering the characteristics of depressive symptoms, occupation, age, work pattern, socioeconomic issues, and other factors must also be taken into account.

This study shows significant correlations between serum vitamin D deficiency and depressive symptoms. The results were significant even after adjusting for shift work, education level, age, and marital status, and the odds ratio of the serum vitamin D deficiency group falling into the depressive symptom group was also significant, at 1.55 (95 % CI = 1.15–2.07). These findings are similar to the results of previous international studies that have been recently reported. In the European Male Ageing Study (EMAS), which used a cross-sectional design, the serum vitamin D concentration showed a negative correlation with depressive symptom [35]. In the 6-year follow-up study of the serum vitamin D level in 954 senior European male subjects, it was found that the higher the serum vitamin D concentration, the significantly lower the CES-D score [36]. In a 9-year prospective cohort study of subjects with cardiovascular disease in the United States, the group with a serum vitamin D level less than 15 ng/mL had a 2.8-fold higher risk of experiencing depression than the group with a serum vitamin D level of 50 ng/mL or above, and this difference was significant [23].

To date, no clear biological mechanisms have been identified to explain the association between serum vitamin D and risk of depression; however, previous international studies have proposed several possible mechanisms. Vitamin D receptors and vitamin D are widely present in brain tissue as well as the central nervous system, and previous studies have reported that these receptors can directly affect the activation of neurons and functioning of the neuroendocrine system [37–39]. Additionally, studies have reported that the receptor genes for various forms of vitamin D that can be synthesized in the body are associated with cognitive impairment as well as depressive symptoms [40], that vitamin D regulates the gene expression of nerve growth factor, which has an important effect on neurotransmission [41], and that vitamin D can act as a neuroprotective factor in suppressing the oxidation and denaturation of neurons through its antioxidant activity [42, 43]. One research group has also speculated that vitamin D may be associated with human cell membrane permeability and nerve conduction in the axon and therefore indirectly regulates neurotransmission [24].

In this study, other variables that had significant correlations with depressive symptoms after adjustment were shift work and education level. The shift work group showed a significant correlation with depressive symptoms (OR = 1.83, 95 % CI = 1.18–2.82) after adjustment, and this finding was similar to those of previous studies on shift work and depression. In a study on depression, anxiety, and immune functioning of shift workers [44] and in another study on the factors related to depression in police officers with either rotating shift work or daytime fixed work schedules [33], shift work was shown to have an effect on biorhythms and was associated with sleeping disorders or depression. Education level also had a significant correlation (OR = 1.39, 95 % CI = 1.02–1.90) with depressive symptoms in the group with a high school diploma or less. This result was similar to those of previous studies on education level and depression. In a study involving a multilevel analysis of the factors influencing depressive symptoms, low education level and depressive symptoms had a significant correlation [45].

This study has several potential limitations. Because this study has a cross-sectional design, it can identify the association between serum vitamin D and depressive symptoms; however, this study cannot explain the exact causal relationship among the variables. Additionally, although a structured questionnaire was used, the possibility remains that individual bias may have influenced the results because it was self-administered. Female electronics-manufacturing indoor workers were selected as subjects of this study, but neither their individual level of outdoor physical activity nor their dietary habits such as consumption of serum vitamin D supplements were assessed. Nevertheless, the advantages of this study are that because it is a single-institution study, the examiner and the environment were consistent, and all the tests were conducted using the same method. Furthermore, by localizing the subjects within the Busan and Gyeongnam area, the influence of latitude and the weather upon the level of serum vitamin D was reduced, and by conducting the examination in a short period between February 2 and March 7, 2014, the influence of differences in seasons was reduced as well. By choosing indoor workers within the same workplace as subjects, the influence of outdoor activities was also reduced.

## Conclusions

Through this study, we found a high percentage of Korean female workers with serum vitamin D deficiency and found a significant association between serum vitamin D deficiency and depressive symptoms. This study is significant in that amidst a serious widespread deficiency of serum vitamin D, a high prevalence of depressive disorder, and a lack of previous studies investigating the association between serum vitamin D and depressive symptoms in Korean subjects, the study has determined the level of serum vitamin D in a large number of Korean female workers and its association with depressive symptoms. Among the various factors that are related to depressive symptoms, serum vitamin D deficiency alone cannot explain depressive symptoms. However, out of the various factors that affect depressive symptoms, through this study we were able to confirm that serum vitamin D deficiency, too, is associated with depressive symptoms. In the future, if serum vitamin D deficiency checkups and care for management of depressive symptoms are provided in the workplace, we can expect to see improved outcomes in the prevention of depressive symptoms. Additionally, there seems to be a need for a study on the relationship of active management of serum vitamin D to the improvement of depressive symptoms in the subjects of the serum vitamin D deficiency group classified into the depressive symptom group.
